# In-Silico Screening of Lipid-Based Drug Delivery Systems

**DOI:** 10.1007/s11095-020-02955-0

**Published:** 2020-11-23

**Authors:** Joscha Brinkmann, Lara Exner, Christian Luebbert, Gabriele Sadowski

**Affiliations:** TU Dortmund University, Laboratory of Thermodynamics, Emil-Figge-Str. 70, D-44227 Dortmund, Germany

**Keywords:** lipid-based formulations, PC-SAFT, solubility, thermodynamic modeling

## Abstract

**Purpose:**

This work proposes an in-silico screening method for identifying promising formulation candidates in complex lipid-based drug delivery systems (LBDDS).

**Method:**

The approach is based on a minimum amount of experimental data for API solubilites in single excipients. Intermolecular interactions between APIs and excipients as well as between different excipients were accounted for by the Perturbed-Chain Statistical Associating Fluid Theory. The approach was applied to the in-silico screening of lipid-based formulations for ten model APIs (fenofibrate, ibuprofen, praziquantel, carbamazepine, cinnarizine, felodipine, naproxen, indomethacin, griseofulvin and glibenclamide) in mixtures of up to three out of nine excipients (tricaprylin, Capmul MCM, caprylic acid, Capryol™ 90, Lauroglycol™ FCC, Kolliphor TPGS, polyethylene glycol, carbitol and ethanol).

**Results:**

For eight out of the ten investigated model APIs, the solubilities in the final formulations could be enhanced by up to 100 times compared to the solubility in pure tricaprylin. Fenofibrate, ibuprofen, praziquantel, carbamazepine are recommended as type I formulations, whereas cinnarizine and felodipine showed a distinctive solubility gain in type II formulations. Increased solubility was found for naproxen and indomethacin in type IIIb and type IV formulations. The solubility of griseofulvin and glibenclamide could be slightly enhanced in type IIIb formulations. The experimental validation agreed very well with the screening results.

**Conclusion:**

The API solubility individually depends on the choice of excipients. The proposed in-silico-screening approach allows formulators to quickly determine most-appropriate types of lipid-based formulations for a given API with low experimental effort.

Graphical abstract
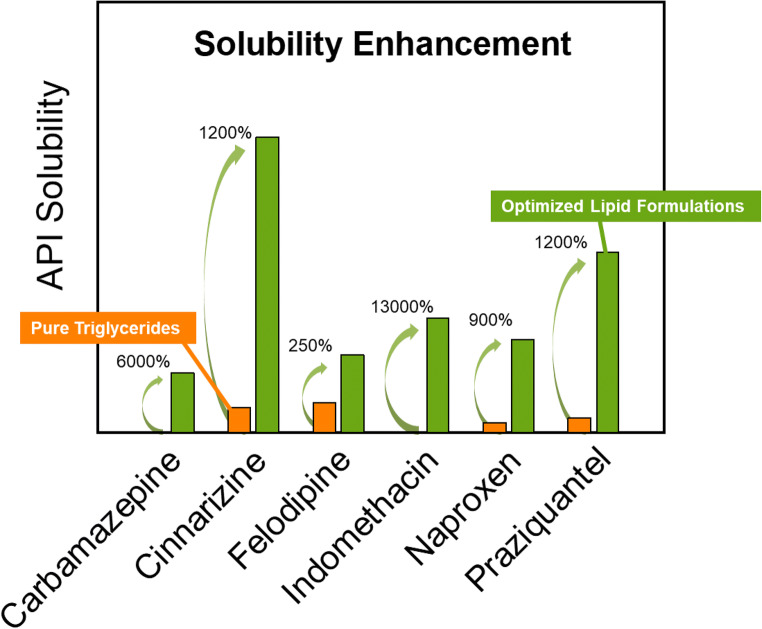

## Introduction

Many of the newly-developed active pharmaceutical ingredients (APIs) possess an insufficient solubility in water. If they cannot be administered as conventional tablets providing sufficient bioavailability, other formulation strategies must be explored to overcome this limitation. Promising alternatives in this field are lipid-based drug delivery systems (LBDDS), in which the APIs are dissolved in liquid formulations. Since the API is administered in a dissolved state, LBDDS can avoid the slow API-dissolution step in the human body and increase the API bioavailability. [1–4] In their simplest form, LBDDS comprise the API dissolved in a pure triglyceride (TG). More often, LBDDS contain complex mixtures of excipients such as glycerides, surfactants or cosolvents to further enhance the API solubility. [5–7].

The API solubility in the liquid excipient (mixture) is a key property for the development of LBDDS. [8] Therefore recent works focused on API-solubility measurements in either TGs [9–11], natural edible oils [11–13], or other commercial excipients [14, 15]. The most-common experimental methods to measure API solubilities are differential scanning calorimetry (DSC) [9, 13, 16], Raman spectroscopy [9, 13], UV-vis [14, 17], and high-performance liquid chromatography (HPLC) [9, 13, 15].

Guidelines to develop LBDDS were discussed in recent works e.g. by Holm [8], Kuentz [5] and Williams *et al*. [18]. According to these works, the first step in the development of an LBDDS is to find out whether or not an API is highly soluble in excipients or mixtures thereof. The large number of commercially available excipients yields numerous combinatory possibilities to formulate an API in a LBDDS.

Pouton [6, 7] developed a lipid formulation classification system (LFCS), which considers different aspects such as aqueous dispersion, digestion and absorption behavior, and suggested promising formulation compositions for further in vitro and in vivo tests. [7, 19] Although the LFCS helps formulators to roughly orientate in the wide field of LBDDS formulations, finding an appropriate excipient mixture for LBDDS still requires countless trial-and-error experiments and is therefore very time consuming and expensive. Thus, there is a strong demand for predictive in-silico methods, which can drastically reduce the experimental effort.

Along this line, this work proposes applying a thermodynamic model, namely the Perturbed-Chain Statistical Associating Fluid Theory (PC-SAFT), for predictive LBDDS-screening purposes [20]. PC-SAFT has already been successfully applied to model API solubilities in pure TGs [9], in natural edible oils [13], and in other excipients [17, 21]. Based on model parameters for pure APIs and excipients as well as for binary mixtures thereof, an in-silico screening is applied in this work to identify excipient mixtures consisting of up to three different excipients providing the highest API solubility. The in-silico screening is then validated via measuring the API solubilities in the proposed formulations.

## Definition of LBDDS Formulation Windows Using the Lipid Formulation Classification System

The LFCS developed by Pouton [6, 7] is as an empirical framework defining reasonable excipient compositions of a LBDDS. It was considered in this work to ensure that the in-silico solubility screening only comprises reasonable excipient compositions. Table I summarizes the four possible formulation classes according to the LFCS and the utilizable excipient compositions in each formulation class. In this work the term *excipient mixture* describes a mixture of excipients without the API, whereas *formulation* stands for a mixture of API and excipients.

**Table I:** LFCS according to Pouton [6, 7] listing the excipients considered in this work

	Composition of the excipient mixture (wt%)				
	Type I	Type II	Type IIIa	Type IIIb	Type IV
**TGs or mixed MGs and DGs:** TG8_0_8_0_8_0,_ Capmul MCM	100	40–80	40–80	<20	0
**Hydrophobic surfactants (HLB < 12):** Capryol 90, Lauroglycol FCC, MC8_0_	0	20–60	0	0	0–20
**Hydrophilic surfactants (HLB > 12):** TPGS1000	0	0	20–40	20–50	30–80
**Hydrophilic cosolvents:** Carbitol, ethanol, PEG400	0	0	0–40	20–50	0–50

The LFCS categorizes LBDDS into four formulation types, while formulation type III is subdivided into two subclasses. The simplest formulations (type I) comprise only an API dissolved in a TG (e.g. TG8_0_8_0_8_0_) or in a mixture of MGs and DGs (Capmul MCM). If the API solubility in type I excipients is found to be too low, 20–60 wt.% hydrophobic surfactants (Capryol 90, Lauroglycol FCC, MC8_0_) might be added (type II formulation). More hydrophilic APIs not sufficiently dissolvable in type I or type II formulations are candidates for type IIIa and IIIb formulations. These formulations become more hydrophilic and thus comprise high contents of hydrophilic surfactants (TPGS1000) and cosolvents (carbitol, ethanol, polyethylene glycol 400 (PEG400)). Type IV formulations finally do not contain any glycerides anymore but only APIs, surfactants and cosolvents. [6, 7].

## Materials and Methods

### Materials

The APIs fenofibrate (FFB), ibuprofen (IBU), indomethacin (IND) and felodipine (FEL) with purity >98% were purchased from TCI (Tokyo, Japan). Cinnarizine (CIN), griseofulvin (GRI), glibenclamide (GLI) were purchased from Alfa Aesar (Karlsruhe, Germany) possessing a purity >97%. Tricaprylin (TG8_0_8_0_8_0_) and carbitol were purchased from Sigma Aldrich (Steinheim, Germany) with purity >99%. The glycerides are abbreviated in this work according to [22] (e.g. tricaprylin: TG8_0_8_0_8_0_, monolaurin: MG8_0_: The first two letters define the type of component (TG = triglyceride, DG = diglyceride and MG = monoglyceride. The capital numbers give the number of carbon atoms in each carbon chain and the subscript denotes the number of unsaturated bonds in each carbon chain.

Caprylic acid (MC8_0_) was obtained from Merck (Darmstadt, Germany) with purity 99%. Capryol 90™ (Capryol 90) was provided by Gattefosse (Saint-Priest Cedex, France). Kolliphor® TPGS (TPGS1000) was provided by BASF (Ludwigshafen, Germany). The surfactants Capryol 90 and TPGS1000 were taken from the same batches as in a recent work [17]. Water was purified by Milli-Q from Merck Millipore (Darmstadt, Germany). Acetonitrile used for the mobile phase in the HPLC was of analytical grade with minimal purity of 99.9% from VWR Chemicals (Darmstadt, Germany). Phosphoric acid was purchased from Sigma Aldrich with purity >99.9%.

### Experimental Methods

The API solubility in an excipient or excipient mixture at given temperature (temperature accuracy ±0.1 K) was determined by equilibrating an excess amount of API in the respective excipient or excipient mixture for at least three days. The saturated liquid phase was then filtered to remove API crystals and diluted with acetonitrile to prevent API crystallization during further handling. The API content in the saturated liquid phase was analyzed with HPLC. An Agilent 1200 HPLC (Santa Clara, USA) with a ZOBRAX Eclipse XDB-C18 reversed-phase column from Agilent (Santa, Clara) was used for this purpose. The column temperature was 35 °C. An acetonitrile/water mixture (70/30 *v*/v) with phosphoric acid (pH = 2.5) was utilized as the mobile phase. The mobile phase had a flow rate of 1 mL min^−1^. All APIs were quantified by a UV-vis detector. FFB, and GLI were analyzed at a wavelength of 286 nm; IBU, FEL, IND, PZQ, CIN, and GRI were analyzed at a wavelength of 225 nm. The excipients were checked not to overlap the UV-vis signal of the APIs at these wavelengths. All samples were measured in triplicates in the HPLC.

The API solubilities were determined in two independent experiments with an averaged uncertainty of 0.64%.

### Solubility Modeling with PC-SAFT

The solubility of a solute i (for instance an API) in glycerides or other excipients is calculated using Eq. 1. [23]

1 $${x}_i=\frac{1}{\gamma_i}\cdotp \exp \left[-\frac{\mathit{\Delta} {h}_i^{SL}}{R\cdotp T}\cdotp \left(1-\frac{T}{T_i^{SL}}\right)-\frac{\mathit{\Delta} {c}_{pi}^{SL}}{R}\left[\ln \left(\frac{T_i^{SL}}{T}\ \right)-\frac{T_i^{SL}}{T}+1\right]\right]$$

Here, *x*_*API*_ is the mole-fraction solubility of the API in the liquid phase. The API activity coefficient *γ*_*API*_ considers all intermolecular interactions among the solute i and glycerides/excipients and was calculated in this work using PC-SAFT. The melting properties of the solute are the melting temperature (${T}_{API}^{SL}$), the melting enthalpy ($\mathit{\Delta} {h}_{API}^{SL}$), and the difference of the heat capacities of the solid and liquid API ($\mathit{\Delta} {c}_{p, API}^{SL}$). Table II contains the melting properties of all APIs considered in this work.

**Table II:** Melting temperature, melting enthalpy and heat capacity difference between liquid and crystalline state of APIs and TPGS1000

Component	${T}_i^{SL}$ [K]	$\mathit{\Delta} {h}_i^{SL}$ [KJ mol^−1^]	$\mathit{\Delta} {c}_{p,i}^{SL}$ [J mol^−1^ K^−1^]	Source
FFB	354.0	33.5	124.3	[9, 24]
IBU	350.2	25.5	50.3	[25]
IND	433.3	39.3	117.0	[21]
CBZ	448.0	26.8	65.2	[26]
GRI	491.2	32.8	93.8	[27]
GLI	446.5	52.9	153.6	[28]
NAP	429.5	31.5	87.4	[28]
CIN	394.0	37.1	113.6	[29]
PZQ	411.5	28.4	103.3	[Unpublished]
FEL	416.9	30.8	89.9	[25]
TPGS1000	311.7	156.2	–	[Unpublished]

PC-SAFT approximates a molecule as a chain of m_seg_ spherical segments possessing a defined diameter (σ_seg_). Dispersive van-der-Waals forces between segments of different molecules are accounted by the dispersive-energy parameter u_i_ k_B_^−1^ (k_B_ is the Boltzmann constant). The association energy parameter ε_i_^AiBi^ k_B_^−1^ and the association volume κ^AiBi^ consider associative forces (e.g. hydrogen bonding) between molecules. Thus, up to five parameters are used to describe the behavior of a pure component, e.g. API or excipient [30]. The pure component parameters used in this work were already available and are summarized in Tables III and IV.

**Table III:** PC-SAFT pure-component parameters of the APIs investigated in this work

API	M	m_seg_	σ_seg_	u_i_ k_B_^−1^	ε_i_^AiBi^ k_B_^−1^	κ^AiBi^	N_assoc_	Source
	[g mol^−1^]	[−]	[Å]	[K]	[K]	[−]	[−]	
FFB	360.80	3.859	4.767	244.8	0.0	0.02	0/2	[9]
IBU	206.28	2.522	4.432	374.7	879.4	0.03	2/2	[31]
IND	357.79	14.283	3.535	262.8	886.4	0.02	3/3	[21]
CBZ	236.27	9.978	2.658	151.6	1094.1	0.02	1/1	[26]
GRI	352.77	14.174	3.372	221.3	1985.5	0.02	2/2	[27]
GLI	494.00	18.278	3.058	221.1	2181.9	0.02	3/3	[28]
NAP	230.26	8.110	2.939	229.5	934.2	0.02	2/2	[28]
CIN	368.51	13.561	3.086	231.0	983.4	0.02	1/1	[29]
PZQ	312.41	6.221	4.090	327.1	0.0	0.02	0/2	[Unpublished]
FEL	384.26	11.528	3.205	234.5	1581.1	0.02	2/2	[25]

**Table IV:** PC-SAFT pure-component parameters of excipients investigated in this work

	M	m_seg_	σ_seg_	u_i_ k_B_^−1^	ε_i_^AiBi^ k_B_^−1^	κ^AiBi^	N_assoc_	Source
	[g mol^−1^]	[−]	[Å]	[K]	[K]	[−]	[−]	
TG8_0_8_0_8_0_	470.69	9.482	4.235	281.6	0	0.020	3/3	[22]
DG10_0_10_0_	400.60	10.965	3.767	242.1	4167.5	0.010	1/1	[Unpublished]
MG12_0_	274.40	7.931	3.738	235.8	3475.4	0.010	2/2	[Unpublished]
MG8_0_	218.29	5.107	3.809	236.2	3475.4	0.010	2/2	[Unpublished]
MC8_0_	144.21	5.306	3.505	255.9	2635.3	0.015	1/1	[Unpublished]
Lauroglycol FCC	258.40	6.848	3.932	290.3	2092.8	0.020	1/1	[17]
Capryol 90	220.31	6.369	3.714	275.8	1405.8	0.020	1/1	[17]
TPGS1000	1513.00	50.988	3.421	254.4	3073.0	0.020	1/1	[17]
carbitol	134.18	4.883	3.387	239.2	2360.5	0.019	1/1	[Unpublished]
PEG400	400.00	20.240	2.899	204.6	1799.8	0.020	1/1	[32]
ethanol	46.07	2.3827	3.177	198.2	2653.4	0.032	1/1	[30]

The segment diameter and the dispersion energy in mixtures are calculated using the Berthelot-Lorentz combining rules (Eqs. 2 and 3).

2 $${\upsigma}_{ij}=\frac{1}{2}\ \left({\upsigma}_i+{\upsigma}_j\right)$$

3 $${u}_{ij}=\left(1-{k}_{ij}\right)\cdotp \sqrt{u_i\cdotp {u}_j}$$

In Eq. 3, a binary interaction parameter (*k*_*ij*_) is introduced when calculating the dispersion energy. It corrects for deviations from the postulated mixing rules for interactions between unlike segments [20]. The *k*_*ij*_-value may linearly depend on temperature (Eq. 4).

4 $${k}_{ij}(T)={k}_{ij, slope}\cdotp T\left[K\right]+{k}_{ij,\mathit{\operatorname{int}}}$$

Binary interaction parameters *k*_*ij*_ used in this work were taken from literature and are summarized in Tables V and VI.

**Table V:** Binary PC-SAFT interaction parameters between excipients and APIs

	FFB		IBU		IND		CBZ		GRI	
	*k*_*ij*, *int*_ [−]	*k*_*ij*, *slope*_ [T^−1^]	*k*_*ij*, *int*_ [−]	*k*_*ij*, *slope*_ [T^−1^]	*k*_*ij*, *int*_ [−]	*k*_*ij*, *slope*_ [T^−1^]	*k*_*ij*, *int*_ [−]	*k*_*ij*, *slope*_ [T^−1^]	*k*_*ij*, *int*_ [−]	*k*_*ij*, *slope*_ [T^−1^]
TG8_0_8_0_8_0_	−0.107 [9]	0.00030	−0.1370 [9]	0.00040	0.024 [9]	0	0.175 *	0	0.031 *	0
DG10_0_10_0_	−0.032 *	0	0.028 *	0	−0.013 *	0	0.040 *	0	0.020 *	0
MG8_0_	−0.032 *	0	0.028 *	0	−0.013 *	0	0.040 *	0	0.020 *	0
MC8_0_	−0.113 *	0.00028	0.027 *	0	0.008 *	0	0.028 *	0	0.028 *	0
Lauroglycol FCC	−0.038 *	0	0.007 *	0	0.011 *	0	0.097 *	0	0.039 *	0
Capryol 90	−0.034 *	0	0.003 *	0	0.003 *	0	0.064 *	0	0.025 *	0
TPGS1000	−0.030 *	0	0.006 *	0	−0.014 *	0	0.023 *	0	−0.007 *	0
carbitol	−0.013 *	0	0	0	−0.009 *	0	0.029 *	0	0.018 *	0
PEG400	0.028 *	0	0	0	0.004 *	0	−0.012 *	0	0.011 *	0
ethanol	0.033 [9]	0	0.1458 *	−0.00030	0.0309 [33]	−0.00008	0.079 [34]	−0.00015	0.032 *	0
	GLI		NAP		CIN		PZQ		FEL	
	*k*_*ij*, *int*_ [−]	*k*_*ij*, *slope*_ [T^−1^]	*k*_*ij*, *int*_ [−]	*k*_*ij*, *slope*_ [T^−1^]	*k*_*ij*, *int*_ [−]	*k*_*ij*, *slope*_ [T^−1^]	*k*_*ij*, *int*_ [−]	*k*_*ij*, *slope*_ [T^−1^]	*k*_*ij*, *int*_ [−]	*k*_*ij*, *slope*_ [T^−1^]
TG8_0_8_0_8_0_	0.024 *	0	0.041 *	0	0.026 *	0	0.017 *	0	0.007 *	0
DG10_0_10_0_	0.010 *	0	0.008 *	0	−0.006 *	0	−0.039 *	0	0.000 *	0
MG8_0_	0.010 *	0	0.008 *	0	−0.006 *	0	−0.039 *	0	0.000 *	0
MC8_0_	0.033 *	0	0.018 *	0	−0.034 *	0	0.000 *	0	0.024 *	0
Lauroglycol FCC	0.044 *	0	0.034 [17]	0	0.026 *	0	0.005 *	0	0.019 *	0
Capryol 90	0.023 *	0	0.013 [17]	0	0.019 *	0	0.007 *	0	0.005 *	0
TPGS1000	−0.011 *	0	−0.003 [17]	0	0.005 *	0	0.023 *	0	−0.016 *	0
carbitol	0.008 *	0	−0.011 *	0	0.006 *	0	0.014 *	0	0.003 *	0
PEG400	0.000 *	0	−0.017 *	0	0.010 *	0	0.058 *	0	0.011 *	0
ethanol	−0.024 [35]	0.00013	−0.001 [33]	0	0.004 *	0	0.020 *	0	0.002 *	0

*The supporting experimental data can be obtained on request

**Table VI:** Binary PC-SAFT interaction parameter *k*_*ij*_ in between the excipients TPGS1000, TG8_0_8_0_8_0_ and carbitol [Unpublished]

Component i/j	TPGS1000	TG8_0_8_0_8_0_	carbitol	ethanol
TPGS1000		0.0120	−0.0100	0.0000
TG8_0_8_0_8_0_			0.0235	0.0300

## Screening Results

All screening results obtained in this work for API solubilities in formulations are full predictions based on the PC-SAFT parameters obtained for pure components and binary systems (see previous section). The screening was performed by comparing the PC-SAFT-predicted API solubilities in excipient mixtures of different formulation types (Table I). A more-complex formulation type was only recommended, when leading to a remarkable increase in API solubility compared to a less-complex one.

### Type I Formulations

**Fig. 1:**
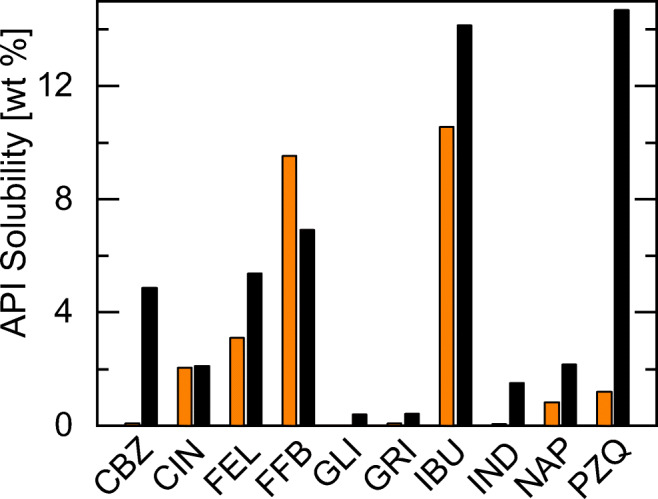
PC-SAFT API solubilities in type I formulations at 25 °C. The left bars are API solubilities in TG8_0_8_0_8_0_, the right bars are API solubilities in Capmul MCM.

Type I formulations are the simplest LFCS formulations. They comprise mixtures of APIs and glycerides. Figure 1 shows the PC-SAFT calculated solubilities of the APIs investigated in this work in TG8_0_8_0_8_0_ and Capmul MCM at 25 °C. The API solubilities in TG8_0_8_0_8_0_ were calculated using the binary parameters from Table V. Capmul MCM is a mixture of different MGs and DGs (mainly MG8_0_, with lower amounts of MG10_0_, DG8_0_8_0_ and DG10_0_10_0_). Due to the complex composition of Capmul MCM, which might differ from batch to batch, the most-prominent components were used for the modeling [13]. Thus, in this work, Capmul MCM was assumed to contain 90 wt% monocaprylin (MG8_0_) and 10 wt% dicaprin (DG10_0_10_0_).

The solubilities of FFB (9.5 wt%) and IBU (10.6 wt%) in TG8_0_8_0_8_0_ were calculated to be high compared to the ones of the other APIs. CIN, FEL and PZQ have solubilities between 1.1 wt% and 3.2 wt% whereas the solubility of CBZ, GLI, GRI, IND, and NAP is even below 1 wt%. These low solubilities make the last-mentioned APIs unfavorable to be formulated in pure TG8_0_8_0_8_0_.

The predicted solubilities of CBZ and PZQ in Capmul MCM are very high compared to those in TG8_0_8_0_8_0_. They are increased by 6000% (CBZ) and 1200% (PZQ) compared to the ones in TG8_0_8_0_8_0_. Also, the solubilities of FEL and IBU in Capmul MCM were predicted to be remarkably enhanced compared to TG8_0_8_0_8_0_. In contrast, the FFB solubility in Capmul MCM was predicted to be 6.9 wt% which is a decrease by 28% compared to the one in TG8_0_8_0_8_0_. For all other APIs, the solubilities in Capmul MCM were predicted to slightly increase compared to the ones in TG8_0_8_0_8_0_, but to not exceed 2.2 wt%.

As a result of this screening step, FFB is recommended to be formulated in TG8_0_8_0_8_0_. The high solubilities of CBZ, FEL, and PZQ in Capmul MCM suggest that they are promising candidates for type I formulations with Capmul MCM or other MG/DG mixtures. IBU reveals high solubilities in TG8_0_8_0_8_0_ as well as in Capmul MCM.

### Type II Formulations

**Fig. 2:**
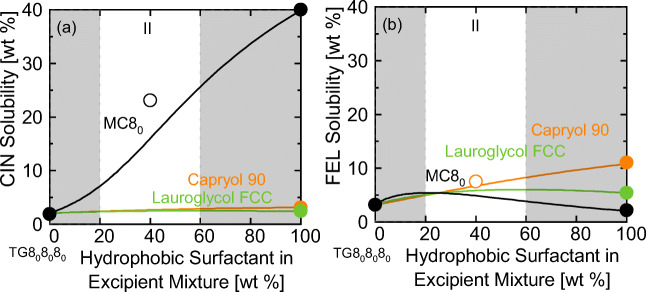
Solubility of (**a**) CIN and (**b**) FEL in mixtures of TG8_0_8_0_8_0_ and either MC8_0_, Capryol 90, or Lauroglycol FCC at 25 °C. Filled circles are experimental data from [Unpublished]. Empty circles are the validation experiments of this work. The white areas are the formulation windows according to Poutons’ LFCS (see Table I).

The solubility of many APIs in type I formulations is quite low (Fig. 1). Thus, the second screening step was to check, whether the addition of a hydrophobic surfactant (type II formulation) can enhance the API solubility in the TG. Figure 2 compares CIN and FEL solubilities in TG8_0_8_0_8_0_ + hydrophobic surfactant mixtures (surfactants being either MC8_0,_ Capryol 90, or Lauroglycol FCC).

The screening results in Fig. 2a show, that the solubility of CIN in TG8_0_8_0_8_0_ strongly increases when adding MC8_0_ to TG8_0_8_0_8_0_ which was experimentally validated for a mixture of 60 wt% TG8_0_8_0_8_0_ and 40 wt% MC8_0_ (CIN solubility of 23.1 wt%). In contrast, the predictions did not reveal a solubility gain upon addition of Capryol 90 or Lauroglycol FCC to TG8_0_8_0_8_0_.

The FEL solubility (Fig. 2b) in TG8_0_8_0_8_0_ was predicted to increase upon addition of Capryol 90. E.g., in an excipient mixture of 60 wt% TG8_0_8_0_8_0_ and 40 wt% Capryol 90, the FEL solubility was predicted to be 6.7 wt%, which very well agrees with the experimental value (7.5 wt%). In contrast to that, the screening suggests that addition of MC8_0_ does not enhance the FEL solubility in TG8_0_8_0_8_0_.

**Fig. 3:**
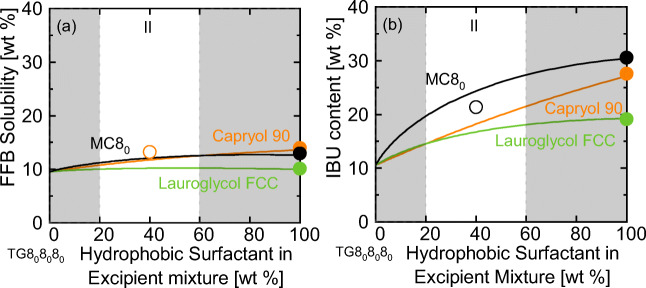
API solubility of (**a**) FFB and (**b**) IBU in mixtures of TG8_0_8_0_8_0_ and MC8_0_, Capryol 90, or Lauroglycol FCC at 25 °C. Filled circles are experimental values measured with HPLC [Unpublished].Empty circles are the validation experiments of this work. The white areas mark Poutons’ LFCS formulation window (see Table I).

FFB and IBU already revealed high solubilities in TG8_0_8_0_8_0_ (Fig. 1). Still, it was investigated whether an addition of a hydrophobic surfactant could further enhance the solubilities of these APIs. Figure 3 thus displays the predicted solubilities of FFB and IBU in mixtures of TG8_0_8_0_8_0_ and one of the hydrophobic surfactants Capryol 90, MC8_0_ or Lauroglycol FCC.

The screening results from Fig. 3a clearly show that the FFB solubility is predicted to only slightly increase when adding hydrophobic surfactants to TG8_0_8_0_8_0_. For adding Lauroglycol FCC, even a slight decrease in FFB solubility was predicted. The experimental FFB solubility in an excipient mixture of 60 wt% TG8_0_8_0_8_0_ and 40 wt% Capryol 90 was afterwards determined to be 13.2 wt% and very well agrees with the prediction.

The screening for IBU (Fig. 3b) shows a distinctively higher solubility in a mixture containing MC8_0_ or Capryol 90 compared to the IBU solubility in pure TG8_0_8_0_8_0_. The predicted IBU solubility for an excipient mixture of 60 wt% TG8_0_8_0_8_0_ and 40 wt% MC8_0_ is 24.3 wt%. The experimental validation revealed an IBU solubility of 21.3 wt%, which again very well agrees with the screening result.

**Fig. 4:**
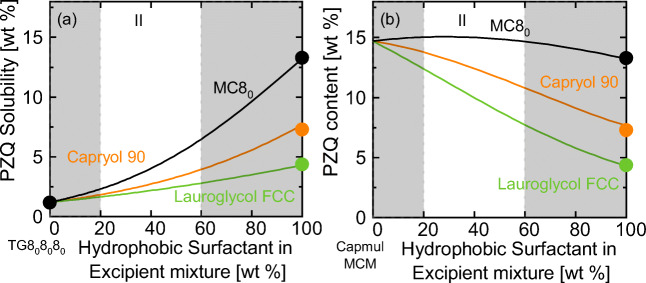
PZQ solubility in mixtures of (**a**) TG8_0_8_0_8_0_/MC8_0_, TG8_0_8_0_8_0_/Capryol 90 and TG8_0_8_0_8_0_/Lauroglycol FCC and (**b**) Capmul MCM/MC8_0_, Capmul MCM/Capryol 90 and Capmul MCM/Lauroglycol FCC at 25 °C. Symbols are experimental literature values measured with HPLC [Unpublished]. The white areas mark Poutons’ LFCS formulation window (see Table I)

The PZQ solubilities in TG8_0_8_0_8_0_ and in Capmul MCM differ remarkably (see Fig. 1). Figure 4 now compares the screening results for the PZQ solubility in type II formulations based on (a) TG8_0_8_0_8_0_ and (b) Capmul MCM.

The low PZQ solubility in TG8_0_8_0_8_0_ was predicted to increase upon addition of any of the investigated hydrophobic surfactants (Fig. 4a). The highest solubility was predicted for excipient mixtures containing TG8_0_8_0_8_0_ and MC8_0_ whereas it was found to be lowest in excipient mixtures of TG8_0_8_0_8_0_ and Lauroglycol FCC. Noticeably, all solubilities screened in Fig. 4a are lower than the PZQ solubility in pure Capmul MCM (Figs. 1 and 4b). Moreover, it can be seen that addition of hydrophobic surfactants to Capmul MCM was predicted to even decrease the PZQ solubility (Fig. 4b).

For all investigated APIs, the solubility was found to be lower in type II formulations containing Lauroglycol FCC compared to those containing Capryol 90. It can thus be concluded that Capryol 90 is the preferred excipient in mixtures with TG8_0_8_0_8_0_ and Capmul MCM as already postulated in an earlier work [17].

Moreover, the screening results revealed that CIN should be formulated in a mixture of TG8_0_8_0_8_0_ and MC8_0_. FEL solubility is highest in an excipient mixture of TG8_0_8_0_8_0_ and Capryol 90. Thus, both APIs are promising candidates for type II formulations. FFB and IBU are sufficiently soluble in every single excipient, even in pure TG8_0_8_0_8_0_ (see Fig. 1) and therefore should be preferably formulated as type I formulations. Only if the solubility of IBU in TG8_0_8_0_8_0_ needs to be further increased, this could be achieved by adding MC8_0_ or Capryol 90. PZQ is suggested to be formulated in mixtures of MGs, DGs and carboxylic acids.

### Type III Formulations

Some APIs show low solubilities in type I and type II formulations. For instance, the screening revealed that the IND solubility does not exceed 2 wt.% in formulations of type I or II. Thus, it was reasonable to proceed the screening for this API and to predict IND solubilities in type IIIa and IIIb formulations. According to the LFCS, these formulation types contain high amounts of hydrophilic surfactant and cosolvent and lower amounts of glycerides (see Table I). The LFCS formulation window shown in the diagram visualizes reasonable compositions for the in-silico screening.

**Fig. 5:**
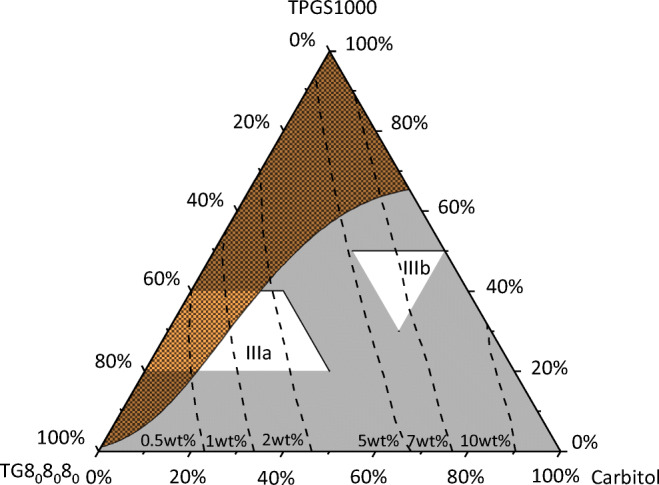
IND solubility in TG8_0_8_0_8_0_/TPGS1000/carbitol mixtures at 25 °C. The squared area is the TPGS1000 crystallization area. Dashed lines are PC-SAFT predictions of constant IND solubility. White areas mark the formulation windows for LFCS type IIIa and IIIb formulations (see Table I).

Figure 5 shows the predicted IND solubilities in a mixture of TG8_0_8_0_8_0_, TPGS1000 (hydrophilic surfactant), and carbitol (cosolvent) at 25 °C. The solubility in pure TG8_0_8_0_8_0_ is quite low (0.1 wt%) and increases to more than 10 wt% for higher amounts of carbitol. Besides the API, TPGS1000 can crystallize at 25 °C. This was considered here and is also visualized in Fig. 5. The region where TPGS1000 is predicted to crystallize in the excipient mixture intersects with the concentration window of type IIIa formulations. In this area, the excipient mixture is not completely liquid and should therefore not be considered for an LBDDS.

The screening results suggest that the IND solubility in type IIIa formulations is considerably lower than in Type IIIb formulations which moreover will not suffer from TPGS1000 crystallization throughout the entire formulation window. Thus, for IND, type IIIb formulations are more promising than IIIa formulations.

### Type IV Formulations

Type IV formulations are considered for those APIs where the addition of glycerides like TG8_0_8_0_8_0_ to the excipient mixture leads to a decrease in API solubility. Formulations of this type do not contain any lipid, but a mixture of hydrophilic and hydrophobic surfactants and cosolvents (Table I). According to Fig. 5, the maximum IND solubility was predicted to be 9.3 wt% in a TG-free mixture of 50 wt% TPGS1000 and 50 wt% carbitol (in perfect agreement with the experimental validation of 10.2 wt%). This is a solubility gain of more than 9000% compared to the IND solubility in pure TG8_0_8_0_8_0_. For this example, it was checked whether the addition of Capryol 90 to an excipient mixture of TPGS1000 and carbitol could further enhance the IND solubility.

**Fig. 6:**
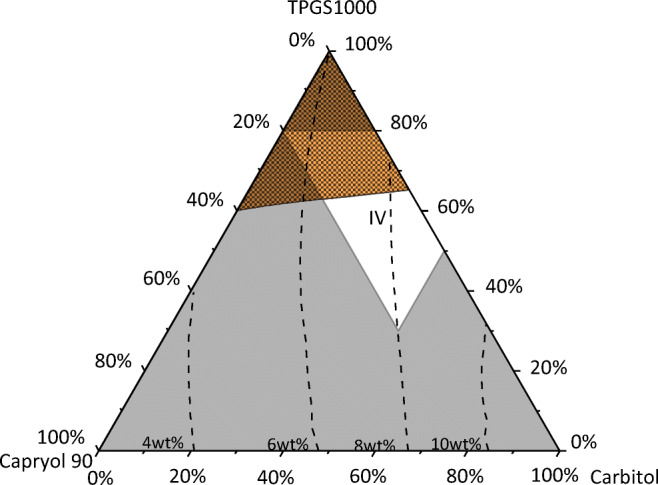
IND solubility in TG8_0_8_0_8_0_/TPGS1000/carbitol mixtures at 25 °C. The squared area is the region where the TPGS1000 crystallizes. Dashed lines are PC-SAFT predictions of constant IND solubility. The white area marks the formulation window for LFCS type IV formulations (see Table I).

Figure 6 shows the predicted IND solubilities in the ternary excipient mixture comprising Capryol 90 (hydrophobic surfactant), TPGS1000 (hydrophilic surfactant) and carbitol (cosolvent) together with the concentration boundaries of type IV formulations as well as the area of TPGS1000 crystallization.

The boundaries of the type IV-formulation window were again found to intersect with the TPGS1000-crystallization region. Thus, the TPGS1000 concentration in the excipient mixtures should not exceed 60 wt% to avoid crystallization. The IND solubility was predicted to increase with increasing amounts of carbitol to up to 9.3 wt% in an excipient mixture of 50 wt% TPGS1000 and 50 wt% carbitol. It can thus be concluded that an addition of Capryol 90 decreases the IND solubility in formulations with TPGS1000 and carbitol. However, it was predicted that the solubility decrease upon addition of Capryol 90 is less pronounced than the decrease upon addition of TG8_0_8_0_8_0_ (compare Fig. 5).

**Fig. 7:**
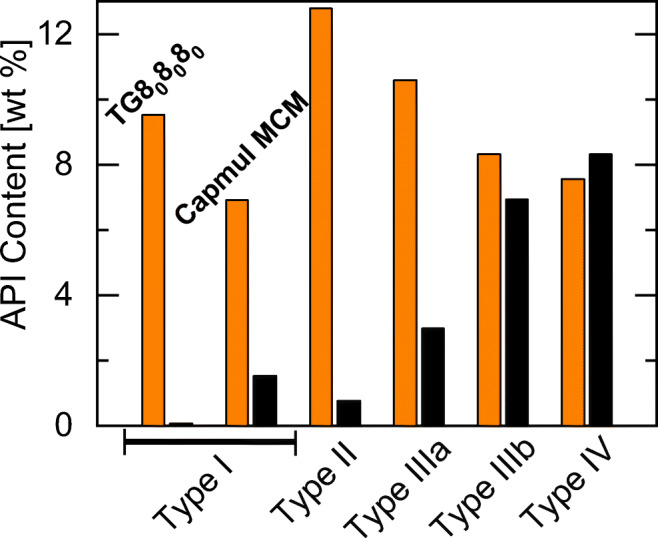
PC-SAFT predicted solubility of FFB (left bars) and IND (right bars) in the different LFCS formulation types at 25 °C. Excipient mixtures contain: pure TG8_0_8_0_8_0_ or Capmul MCM (type I); 40 wt% TG8_0_8_0_8_0_ and 60 wt% Capryol 90 (type II), 40 wt% TG8_0_8_0_8_0_, 20 wt% TPGS1000, and 40 wt% carbitol (type IIIa); 15 wt% TG8_0_8_0_8_0_, 35 wt% TPGS1000, and 50 wt% carbitol (type IIIb), 15 wt% Capryol 90, 35 wt% TPGS1000, and 50 wt% carbitol (type IV).

Figure 7 compares the solubilities of FFB and IND in all considered formulation types (types I to IV). It clearly shows that the screening results for FFB and IND are very different for the different formulations types of the LFCS. The solubility of IND is predicted to be very low in type I and II formulations and is distinctively enhanced in type III and IV formulations. In contrast, the predicted FFB solubility is already high in TG8_0_8_0_8_0_ and only slightly increases in the type II formulation. For types III and IV formulations, the FFB solubility is predicted to decrease due to the lower TG8_0_8_0_8_0_ concentrations in the excipient mixtures.

**Fig. 8:**
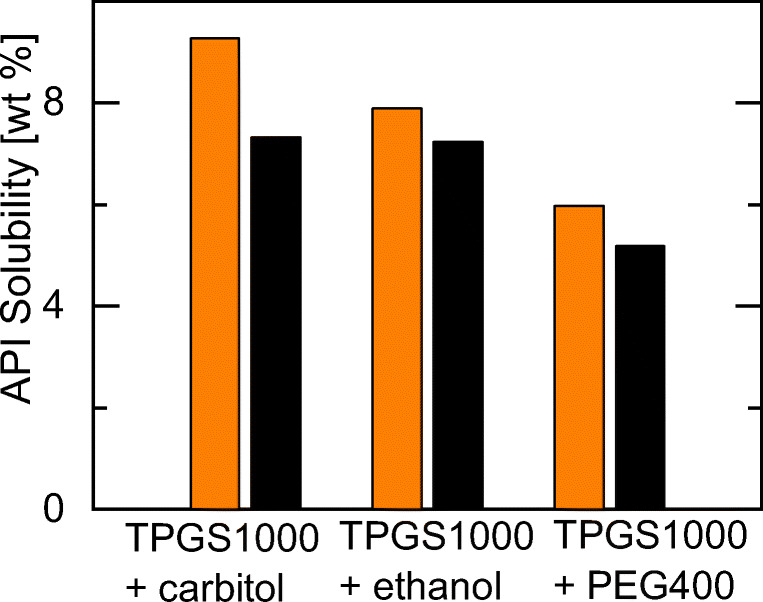
Screening results for the solubility of IND (left bars) and NAP (right bars) in excipient mixtures of 50 wt% TPGS1000 and 50 wt% cosolvent (carbitol, ethanol, PEG400) at 25 °C. API solubilities were predicted with PC-SAFT.

In a next step, the screening approach was extended to investigate whether the solubility of IND and NAP could further be enhanced by choosing another cosolvent than carbitol. Figure 8 compares IND and NAP solubilities in excipient mixtures of 50 wt% TPGS1000 and 50 wt% of either carbitol, ethanol, or PEG400.

The screening results reveal the highest IND and NAP solubilities in an excipient mixture of TPGS1000 and carbitol. For both APIs, the solubility is predicted to be lowest in the excipient mixture of TPGS1000 and PEG400. E.g., the NAP solubility is predicted to decrease from an TPGS1000/carbitol mixture to a TPGS1000/PEG400 mixture by approximately 30%. Thus, carbitol is recommended here as cosolvent in type IIIb or IV formulations for IND and NAP.

These results underline the importance of investigating the influence of different cosolvents. Especially for type IIIb and IV formulations which contain up to 50 wt% cosolvents in the excipient mixture, identifying the most-suitable cosolvent is very important.

### Formulating the Brick-Dust APIs GRI and GLI

**Fig. 9:**
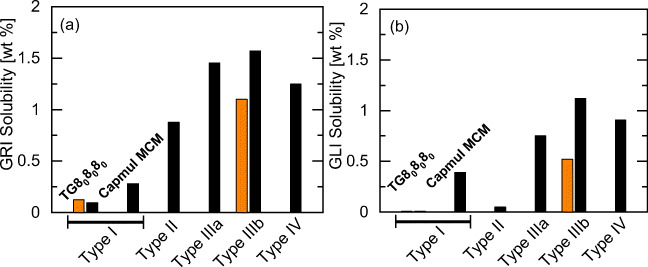
Solubility screening results of (**a**) GRI and (**b**) GLI at 25 °C comparing the solubility in excipient mixtures of each formulation type. Right bars are the PC-SAFT predictions, left bars are validation experiments. The excipient mixtures contain either pure TG8_0_8_0_8_0_ (type I); 60 wt% TG8_0_8_0_8_0_, and 40 wt% MC8_0_ (type II); 40 wt% TG8_0_8_0_8_0_, 20 wt% TPGS1000, and 40 wt% carbitol (type IIIa); 15 wt% TG8_0_8_0_8_0_, 35 wt% TPGS1000, and 50 wt% carbitol (type IIIb), 15 wt% Capryol 90, 35 wt% TPGS1000, and 50 wt% carbitol (type IV).

GRI and GLI are denoted as brick-dust APIs [3] as their solubility in any excipient mixture is extremely low and it is most challenging to find appropriate formulations for these API candidates. Figure 9 summarizes the screening results for GRI and GLI formulations from all LFCS types and the solubility gain, which can be achieved by each of these.

As it can be seen in Fig. 9, the solubility of GRI can distinctively be enhanced by a type IIIb formulation (PC-SAFT: 1.6 wt%) compared to the GRI solubility in type I formulations. The experimental solubility (1.1 wt%) was found in very good agreement with this screening result. Although this value is almost 900% higher than the GRI solubility in pure TG8_0_8_0_8_0_ (0.1 wt%), the GRI solubility is still quite low. No further gain of the GRI solubility was predicted when formulating it in a type IV formulation (PC-SAFT predicted solubility in 15 wt% Capryol 90, 35 wt% TPGS1000 and 50 wt% carbitol: 1.2 wt%). In a recent work, also other excipient mixtures were examined to find higher GRI solubilities [36]. However, among all systems considered in that work, the maximum GRI solubility was only found to be 1.5 wt% which compares to the values found above for the type IIIb formulation.

In case of GLI (Fig. 9b), the predicted solubility for all formulation types is even lower than the one of GRI. As before, the GLI solubility is predicted to increase for more hydrophilic formulation types (IIIb and IV). The experimental solubility in the IIIb formulation was found to be 0.6 wt%, which agrees very well with the predicted ones. Although the GLI solubility in the type IIIb formulation shows a gain of more than 8000% compared to the type I formulation (see Fig. 1), the GLI solubility does not exceed about 1 wt%. However, the experimental GLI solubility of 0.5 wt% might be sufficient for LBDDS applications as the dose in a commercial tablet is only 1 to 5 mg [37]. This dose would be achieved in 1 g LBDDS of type IIIb or IV according to the screening performed in this work as well as its experimental validation.

Table VII lists the logP values of the ten APIs considered in this work as well as the formulation types recommended from the screening in this work. Obviously, we do not observe a correlation between the logP value of the APIs and the recommended formulation type as sometimes discussed in literature [1]. It is thus not reasonable to choose formulation types for APIs based on their logP value only. Instead, it is strongly recommended to screen the solubility of a given API on a case-to-case basis considering the specific interactions between the API and the components of an excipient mixture.

**Table VII:** APIs considered in this work, their logP values and recommended formulation types. Formulation type 0 denotes brick dust APIs

API	logP	formulation type	reference
GRI	2.2	0	[14]
PZQ	2.7	I	[14]
CBZ	2.7	I	[14]
NAP	2.8	IIIb or IV	[14]
FEL	3.6	II	[14]
IBU	4.0	I or II	[38]
GLI	4.1	0	[14]
IND	4.2	IIIb or IV	[14]
FFB	5.1	I	[14]
CIN	5.5	II	[14]

## Conclusions

An in-silico solubility screening of ten model APIs was conducted to identify appropriate excipient mixtures for lipid-based drug delivery systems according to the lipid-based classification system developed by Pouton. Using this screening approach, promising formulation types for eight out of the ten model APIs were found, namely

- Type I: fenofibrate, ibuprofen, praziquantel and carbamazepine
- Type II: cinnarizine and felodipine
- Types IIIb and IV: naproxen and indomethacin.

The solubilities of griseofulvin and glibenclamide were also predicted to be best in type IIIb and type IV formulations. However, they remained very low (<1.6 wt%) even in these formulations.

No correlation was found between the logP value of the considered APIs and the recommended formulation type. Obviously just considering logP is not sufficient. Instead, specific molecular interactions between an API and the components of the excipient mixture need to be considered on a case-by-case basis in the development of lipid-based drug delivery systems. Thus, in-silico screening based on thermodynamic modeling is a powerful tool to rapidly determine promising lipid-based drug delivery systems. It can be extended to additional excipients or APIs and thus allows to drastically decrease the number of experiments required for subsequent in vitro or in vivo tests.

## Nomenclature

a: Helmholtz energy J mol^−1^
h: molar enthalpy mJ mol^−1^
c_p_: heat capacity J (mol K)^−1^
M: molar mass g mol^−1^
m: segment number -
k_B_: Boltzmann constant J K^−1^
k_ii_: binary interaction parameter -
N: number of data points -
N_assoc_: number of association sites -
R: ideal gas constant J (mol K)^−1^
p: pressure bar
T: temperature K, °C
u: dispersion energy J
w_i_: mass fraction -
x_i_: mole fraction -

## Greek Characters

γ: activity coefficient -
ε^AiBi^: association energy J.
ρ: density kg m^−3^
κ^AiBi^: association volume -
σ_seg_: segment diameter Å

## Subscripts

i,j: component
int: intersection

## Superscripts

assoc: associating
disp: dispersion
hc: hard chain
L: liquid
res: residual
S: solid
V: vapor
